# Continuum of Care: Positioning of the Virtual Hospital

**DOI:** 10.3389/fcvm.2021.779075

**Published:** 2022-03-15

**Authors:** Anne Catherine M. H. van der Lande, Roderick W. Treskes, Saskia L. M. A. Beeres, Martin J. Schalij

**Affiliations:** ^1^Executive Board, Leiden University Medical Center, Leiden, Netherlands; ^2^Department of Cardiology, Leiden University Medical Center, Leiden, Netherlands

**Keywords:** chronic diseases, eHealth, polypharmacy, continuum of care, virtual care

## Abstract

**Introduction:**

Patients with multiple chronic diseases suffer from reduced life expectancy. Care for these patients is often divided over multiple healthcare professionals. eHealth might help to integrate care for these patients and create a continuum. It is the primary purpose of this paper to describe an intervention that integrates first, second, and third line care in patients with multiple chronic conditions using remote monitoring, remote therapy and data automatization, all integrated in a virtual care center (VCC).

**Methods:**

Patients diagnosed with three or more chronic conditions are included and given smartphone compatible devices for remote monitoring and a tablet for video consultations. Patients will be followed-up by the VCC, consisting of nurses who will coordinate care, supervised by general practitioners and medical specialists. Data is reviewed on a daily basis and patients are contacted on a weekly basis. Review of data is automated by computer algorithms. Patients are contacted in case of outcome abnormalities in the data. Patients can contact the VCC at any time. Follow-up of the study is 1 year.

**Results:**

The primary outcome of this study is the median number of nights admitted to the hospital per patient compared to the hospitalization data 12 months before enrolment. Secondary outcomes include all-cause mortality, event free survival, quality of life and satisfaction with technology and care.

**Conclusion:**

This study presents the concept of a VCC that integrates first, second, and third line care into a virtual ward using remote monitoring and video consultation.

## Introduction

The growing number of patients with multiple chronic diseases is a challenge for healthcare professionals, insurers, and healthcare systems in countries across the globe. There are multiple reasons to explain this growing number. Firstly, there has been a global increase in life expectancy with those in the 60-year age group expected to live an additional 20 years in the last decade (1). Therefore, the older population is increasing rapidly worldwide (2). Secondly, the prevalence of chronic diseases is increasing over the last years. Among people over age of 65 the proportion of individuals with multiple chronic diseases is estimated at about 65% and about 85% among people over age of 85 (3, 4). Research has shown that clinical outcome worsens with each additional chronic disease (5). Patients live shorter with less quality of life. An American study in Veterans found that 5-year mortality rates increased from 4.1% in veterans with no chronic diseases till 16.7% in veterans with four or more chronic conditions (5). These patient populations form a challenge for healthcare systems as well: patients are often treated by multiple doctors in different hospitals and use multiple medications, which increases the chance of contradictory treatment advices and medication errors. One Spanish study found that in 114 patients with multiple chronic diseases, a medication error was found in 75% of cases (6). This results in worse clinical outcomes, as a Swedish study demonstrated that fatal adverse drug reactions form 3% of all deaths. Median age of patients who die from adverse drug reactions (ADR's) was 82 years in that study (7). This chance might be amplified if patients are treated at multiple hospitals with different electronic medical records who are commonly not integrated, increasing chances of asymmetry in up-to-date medication lists between healthcare providers. It might be for these reasons, that patients with multiple chronic diseases are the most costly patients for every healthcare system. In the United States, ~5% of the population accounts for 45% of total healthcare expenditure; this group predominantly consists of patients with multiple chronic diseases (8). Furthermore, it may be beneficial to monitor patients more or less continuously not only in the hospital but separate in their daily live or home situation in order to prevent deterioration.

There is therefore a need for an intervention that improves clinical outcomes in patients with multiple chronic diseases while at the same time lowering costs. It is hypothesized that improving care coordination by centralizing it, as well as continuous monitoring with automated data analysis, might reduce medication errors, improve treatment decisions and therefore improve clinical outcomes and reduce costs.

### Case

To illustrate the rationale of this project, the case of a 78-year old male is presented who is referred to the nephrologist because of worsening renal function. His medical history comprised atrial fibrillation, coronary artery disease, aortic and mitral valve replacement, heart failure and pulmonary hypertension for which he was treated at an academic hospital. He was also known with benign prostate hyperplasia and COPD for which he was treated in two different general hospitals. Furthermore, the general practitioner (GP) treated him for gout and reflux oesophagitis.

He now was referred to the nephrologist in general hospital B since his estimated glomerular filtration rate (eGFR) decreased from 45 to 22 mL/min/1.73 m^2^ in 3 years. The patient reported severe fatigue since 2 weeks, weight loss since 6 months and dyspnea at exercise since multiple years. His walking distance was ~20 m. He reported to take 14 different pills a day among which bumetanide, spironolactone and hydrochlorothiazide. Physical examination showed a pale, cachectic, frail man. Laboratory testing showed a normocytic anemia (Hb 5.2 mmol/L, MCV 82 fL), urea of 44 mmol/L and a creatinine of 232 mmol/L (eGFR 22 mL/min/1.73 m^2^). Ultrasonography showed normal kidney sizes with cortical thinning but no intrarenal congestion.

After written consent of the patient, the cardiologist in the academic hospital sent the patients' medical record by secured mail. It revealed that the patient was not using hydrochlorothiazide anymore. Interestingly, the GPs record noted the use of 12.5 mg daily. As the worsening renal function could be attributed to the sequential nephron block, the hydrochlorothiazide was stopped. Thereafter, eGFR improved from 22 to 37 mL/min/1.73 m^2^ and his urea lowered to 22 mmol/L. This was paralleled by an increased exercise capacity and reduction in fatigue without development of cardiac congestion. This case illustrates that the involvement of multiple different doctors at different locations can jeopardize a patient's health by scattering of medical data and conflicting information on medication. Furthermore, the repeated hospital visits generated discomfort for the patient and unnecessary health care costs for the society.

This case illustrates that scattering of medical information, multiple doctors in different hospitals and multimorbidity with polypharmacy can jeopardize a patient's health. It furthermore illustrates the discomfort a patient might experience of having to be physically present at the outpatient clinic or day care clinic multiple times at different locations.

**Figure 1:**
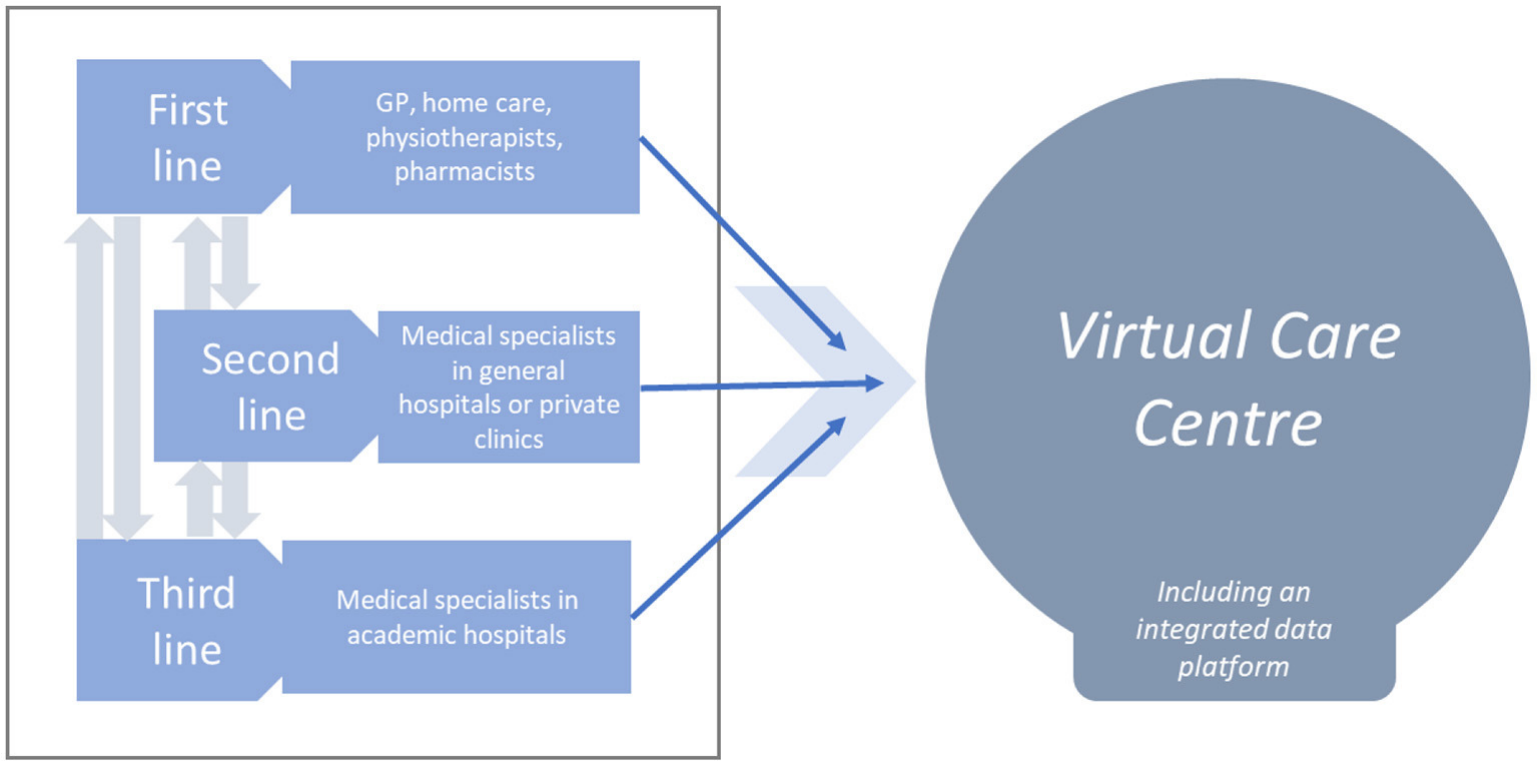
Schematic representation of integrated health care in the virtual care center (VCC).

It is therefore the primary purpose of this paper to describe the rationale and design of an interventional single arm trial with historical comparison to evaluate the effect of integrating first, second, and third line care in patients with multiple chronic conditions using remote monitoring, remote therapy and data automatization, all integrated in a virtual care center (VCC). As the case is an illustration for VCC, the next steps of the patient journey are out of scope. An overview of the integration of the first, second, and third line of care is illustrated in Figure 1.

## Methods

### Patient Population

For this project, patients diagnosed with three or more of the following conditions are considered eligible for inclusion: diabetes mellitus (type one or type two), myocardial infarction, stroke (either ischaemic or haemorrhagic), peripheral arterial disease, hypertension, dementia, heart failure, coronary artery disease, and/or chronic obstructive pulmonary disease. These conditions were chosen because they allow for remote monitoring of data, are prevalent, often coincide and put a significant burden on health care systems.

Patients are excluded from participation if they are unwilling to sign informed consent, if they do not speak Dutch or English at a sufficient level (at least B1), if they do not have internet access, if they are not at least 18 years old at time of inclusion and if they are pregnant or wishing to become pregnant within 1 year after study inclusion. From multiple occasions patients will be evaluated for inclusion.

Possible sites of inclusion are the outpatient clinic, wards, and emergency departments of participating centers. General practitioners who are participating in the project can approach patients for participation as well.

### Design

Patients are approached for participation by their treating general practitioner or medical specialist who will ask them if they want to receive more information about the project. They will receive a phone call from a research assistant < 3 working days who will explain the project in detail. Afterwards, patients are given the opportunity to fill in the informed consent and contemplate participation. If they are willing to participate, a 14 days pilot is started to see if the patient can cope with basic mobile technology, is able and willing to communicate with the medical team and is compliant with medical and technical advice. In this 14 days pilot, patients will only receive a smartphone (or borrow a smartphone device) and blood pressure monitor. Patients can quit the project any moment without giving a reason or having to sign a paper.

After the 14 days pilot, the research team will go to the patients home to install all of the required medical devices, install all home automatics, install the apps for video consultation and to test if everything is working. One family member or acquaintance will be requested (digitally) present during the installation process. Patients are also instructed on the frequency of testing (individualized per patient) and the frequency of data reviewing (daily). Patients are instructed that they can contact the VCC continuously, including in case of a possible emergency. The day after the installation process, all data are send and reviewed with the patient and one family member.

### Devices

The number of devices will be individualized and given to the patient by medical indication. In brief, the following devices can be given to the patient dependent on the chronic condition. An overview of the devices per chronic condition is presented in Table 1.

**Table 1:** Devices per medical indication.

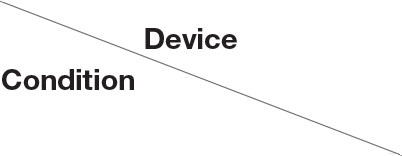	**Heart rate/ ECG monitors**	**Weight scale**	**Blood pressure monitor**	**Activity tracking**	**Home automation**	**Video consultation**	**Spirometer**	**Glucose meter**
Diabetes mellitus (type I or type II)	✓	✓	✓	✓	✓	✓	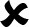	✓
Myocardial infraction	✓	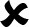	✓	✓	✓	✓	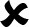	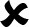
Stroke	✓	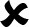	✓	✓	✓	✓	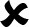	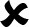
Peripheral arterial disease	✓	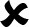	✓	✓	✓	✓	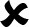	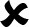
Hypertension	✓	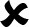	✓	✓	✓	✓	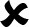	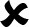
Dementia	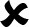	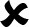	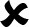	✓	✓	✓	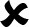	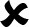
Chronic obstructive pulmonary	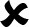	✓	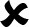	✓	✓	✓	✓	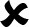
disease (COPD)								
Heart failure	✓	✓	✓	✓	✓	✓	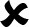	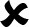
Coronay artery disease	✓	✓	✓	✓	✓	✓	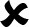	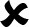

### Heart Rate/ECG Monitors

There are four devices that could be given to the patient for ECG monitoring, namely a 12-lead ECG device (CardioSecur) and/or single lead ECG device (Kardia, Withings Move and Apple Watch) these devices have been described previously (9–13).

### Weight Scale

In case a weight scale is clinically indicated (monitor weight loss, any patient with heart failure, as part of a combined lifestyle intervention), patients will receive a Bluetooth and WiFi enabled weight scale (Withings, Issy-les-Moulineaux, France).

### Blood Pressure Monitor

In case a blood pressure monitor is indicated (follow-up for hypertension, secondary or tertiary prevention of atherosclerosis), patients are given a smartphone compatible oscillometric device (Withings, Issy-les-Moulineaux, France) that is completely automated. Data are automatically stored on the phone and transferred to the hospital.

### Activity Tracking

In case activity tracking is indicated (secondary or tertiary prevention of atherosclerosis or in case of heart failure), patients are provided with a watch that counts both steps per day and sleep duration. Patients who are equipped with an implantable cardioverter defibrillator that is able to check these parameters will not receive an activity tracker.

### Home Automation

Fall detectors will be installed at patient's house to register falls. In case of a potential fall, a nurse from the VCC will visit the patient.

### Spirometry

Patients will be provided with a digital spirometer that is able to measure the forced expiratory volume in one second as a marker for early detection of exacerbation COPD.

### Video Consultation

Patients will be provided with an app *via* which a secure video connection can be made with the VCC. If patients do not own a tablet of smartphone, they will be provided with a loan device. Patients can contact the VCC continuously (“24/7”).

### Technical Support of Devices

At study inclusion, all relevant apps are installed on the device of the patient by a technical assistant of the VCC. If patients do not own a device that is compatible with the necessary apps, they will be provided with a borrowed device. Patients are instructed by the technical assistant of the VCC on how to use devices.

### The Virtual Care Center

Data from home monitoring devices are synchronized four times per day with the data servers of the Virtual Care Center (VCC). These data are reviewed by an algorithm that notifies healthcare professionals in case of deviations from previous trends. These algorithms are developed by IT specialists who are employed by the VCC. Briefly, the algorithm notifies the professional in case a data point exceeds an upper or lower limit and/or deviates more than a previous set limit from the average of 10 previous values. These limits are individualized per patient and are prescribed by the patient's physician. In Table 2 an overview of advantages and disadvantages for all stakeholders when switching to the virtual care center are presented.

**Table 2:** Overview of advantages and disadvantages for all stakeholders when switching to the virtual care center.

**Stakeholder**	**Advantages**	**Disadvantages**
Patients	1) More contact with health care professional(s) 2) More control over health-data 3) Care at home, more independent	1) Various technical devices to manage with risk of user errors
Family	1) More options to help your family member and to check up on results 2) Easy to join a consultation of your relative	1) Various technical devices to help with when needed with risk of user errors
Nurses	1) More variety in work	1) Less physical contact with patients
General practitioners (GPs)	1) More variety in work 2) When tracking chronic patients virtually, more time for more complex patients at the general practice 3) Provide care more efficiently	1) Many patients to supervise 2) More at a distance of the patient, therefore not being able to check visually how the patient is doing
Medical specialists	1) More less complex care at home, therefore more complex care at the hospital 2) Less unnecessary care at the hospital 3) Provide care more efficiently	1) More at a distance of the patient, therefore not being able to check visually how the patient is doing
Healthcare payer	1) Less healthcare costs	1) No existing insurance for virtual care at the moment

**Figure 2:**
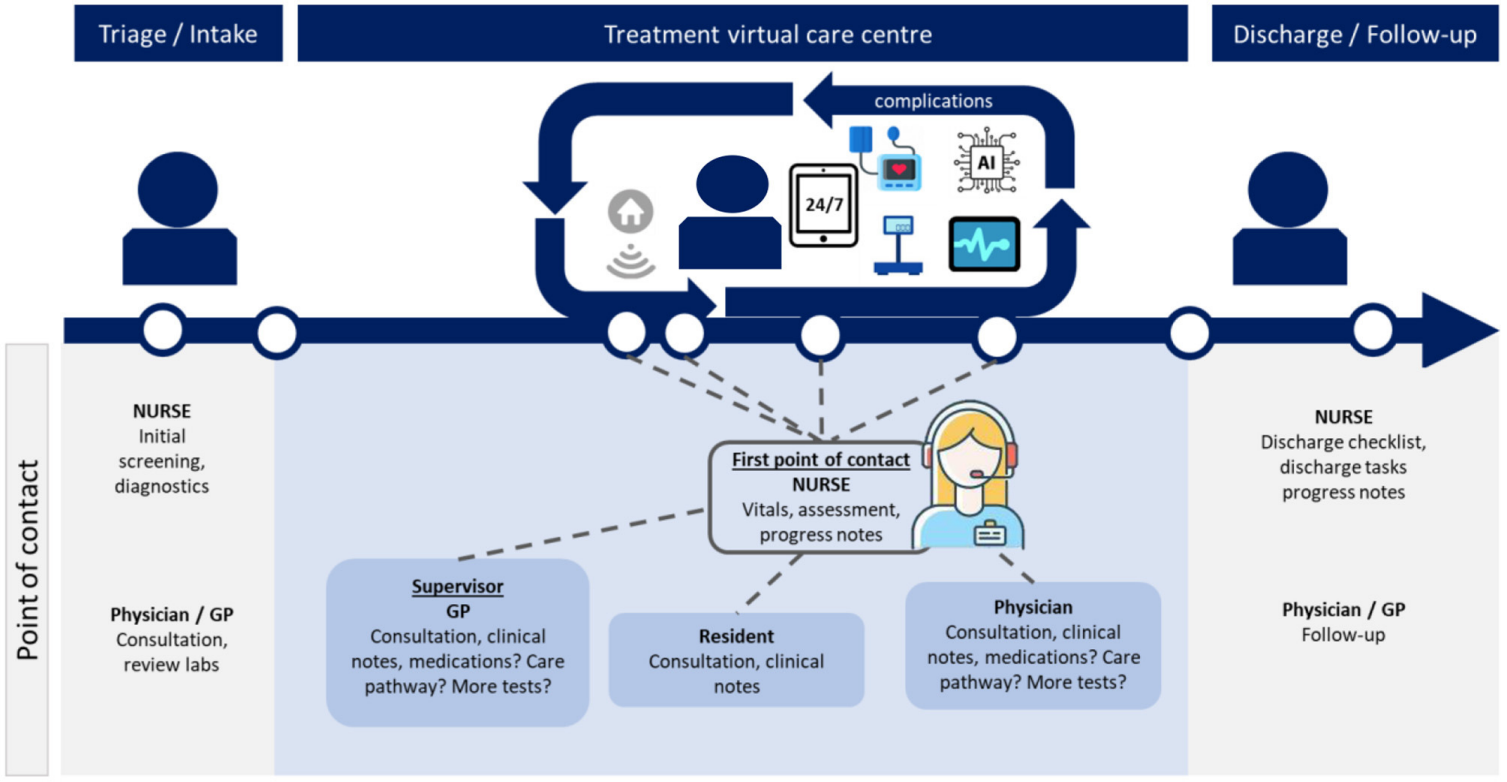
Patient journey virtual care center.

Each patient will keep contact with his/her own nurse who works in the VCC, supervised by a GP. The nurse is trained in recognizing multi-problems. The nurse will request the medical specialist that is needed in consult for the patient in the VCC. The general practitioner will be primarily responsible for adequate treatment of the patient and will be informed at all times. The VCC is furthermore populated by residents, which may be requested in consultation by the nurse when needed. This process is illustrated in Figure 2.

### Follow-Up

Patients who are included are contacted one time per week by their treating physician. Symptoms are inventoried and data are reviewed. This interview is not standardized because individualization is the key of the physician following up the patient for longer time. In case additional testing is needed, this is primarily done at the patient's home. For low complex additional testing (echocardiography, ultrasound of the abdomen, X-ray) of the chest a patient is referred to a allied testing center in the same town the patient is living in. Laboratory testing is performed at home.

### “Discharge” of the Virtual Care Center

Follow-up of the study is 1 year. After 1 year when follow-up is completed, patients can, if they want to, remain for treatment at the VCC. Data will be kept for research purposes.

### Data Reviewing

Data will be reviewed by an algorithm, as described above. Patients can contact the VCC 24 h per day, 7 days per week to discuss their data and whether or not they should take medication.

### Data Integration Into the EMR

Data will be integrated in one platform. In this platform, data is integrated and notes are kept, as well as a summary of the patients medical history. This platform can be accessed by pharmacists, general practitioners, medical specialists who qualify according to Dutch law as having a therapeutic relationship with the patient. Access is logged to identify health care professionals that review data of patients that they are not in a therapeutic relationship with.

### Ethical Conduct

The study will be conducted according to the principles of the Declaration of Helsinki. Written informed consent will be obtained from all patients that participate in the project. For this study, Ethical Approval of the hospital's Medical Ethics Committee will be sought. All devices are CE marked, are approved for sale in Europe and approved for medical use. For this study, no data safety monitoring board will be installed since treatment will not deviate from national or international guidelines.

### Study Withdrawal

Patients can withdraw the study anytime they want without having to give a reason. This is written in the patient information folder. In case patients withdraw from the study, they are registered as lost to follow-up in the database. Municipality registers will be checked after the follow-up period to review if patient's are alive. Patients who withdraw from the study within 1 month after inclusion will not be included in the analysis of the primary endpoint.

### Non-adherence

If a patient is unannounced not replying to messages from the hospital within 24 h, is not adhering to the measurement schedule for more than 5 consecutive days and/or is not replying within 24 h to inquiries from the VCC, the patient is defined as being non-adherent. In case of non-adherence, a meeting with the patient and a family member will be scheduled within 10 working days to investigate patient's reasons for being non-adherent. Patients will be stimulated to become adherent again. If the patient within 1 month again fulfills the criteria for non-adherence, the project for this patient is terminated.

## Results

### Primary Outcome

The primary outcomes of this study are the median number of nights admitted to the hospital per patient as well as the number of hospitalization compared to the hospitalization data 12 months before enrolment. Hospital records (electronic medical records) will be the primary source of data. A night is registered as a night if the patient is admitted before 04.00 h in the morning (i.e., 04.00 ante meridiem, AM). If a patient is admitted after 04.00 AM and discharged the same calendar date, the visit is counted as a day care/short stay visit.

### Secondary Outcomes

A complete list of secondary outcomes including their definitions and how they are measured is given in Table 3. Secondary outcomes include all-cause mortality, event free survival, quality of life, and satisfaction with technology and care.

**Table 3:** Secondary outcomes and their definitions.

**Outcome**	**Definition**	**Measured by**
All-cause mortality	Death from any cause during trial participation	Electronic medical records (EMRs) and/or municipality registers
Event free survival	Time in days until the patient reaches an “outcome,” defined as an unplanned hospitalization, emergency department visit, or death from any cause	EMR
Medication changes	Any chance in dose and/or frequency in prescribed medication or the initiation or termination of a prescription of a daily or weekly taken medicine	EMR
Quality of life	Score derived from EQ-5D	EQ-5D
Number of hospital admissions	A hospital admission is noted if a patient is admitted to the ward, intensive care or cardiac care unit of one of the participating centers and involves a date change. As noted above, if the patient is admitted between 12.00 and 03.00 AM, it is counted as a hospital admission	EMR
Hospital admission duration	Number of nights spent in the hospital	EMR
Emergency department visits	Any visit for a medical problem of which a notation in the EMR is made to one of the emergency departments of participating centers	EMR
Number of in-office outpatient clinic visits	Any visit for a medical problem to one of the outpatient clinic visits of participating centers of which a notation in the EMR is made	EMR
Number of unplanned in-office outpatient clinic visits	An in-office visit is labeled as “unplanned” if the visit is planned <48 h in advance. Rescheduled visits are not labeled as unplanned	EMR
Number of GP visits	Any visit for a medical problem to the GP of which a notation in the EMR is made	EMR

## Discussion

In this paper, the rationale and design of a single arm trial with historical comparison is presented. It is the primary purpose of this study to propose a concept of an intervention that might improve care in patients with multiple chronic diseases.

### Virtual Wards

Virtual wards have been the subject of various pilot trials and a randomized controlled trials of 1,923 patients (2, 14–16). Patient populations and the extensiveness of the intervention differed, which compromises comparisons. Generally speaking, frail elderly patients with multiple chronic conditions were admitted to a virtual ward, where contact with patients was kept *via* telephone or home visits and treatment was adjusted either by physician's discretion or after interprofessional team meetings (2, 14–16). Two pilot studies found a statistically significant reduction in number of hospital admissions and/or emergency department visits (2, 15). The large randomized controlled trial however, found no statistically significant effect on 30-day readmissions in patients who had just been discharged (14).

The study presented in this paper differs from the methods described in the previously published papers on virtual wards. Although this study follows the same basic model, the extensiveness of the intervention and the setting differ considerably. This study uses smartphone compatible devices and home automation to monitor patients at home, contact will be made *via* videoconferencing so that family members can join in. Secondly, previously described initiatives were primarily done from one “line of healthcare,” either by first or second line healthcare. In this study, first, second, and third line care are integrated and present in one virtual care center, thereby easing communication and reducing duplicity of diagnostic testing. Thirdly, treatment with IV medication and basic diagnostic testing will be done at home, thereby hypothetically reducing emergency department visits and hospital admissions. Finally, automated data analysis of continuous data that generates early warning alerts will help to improve early detection of deteriorating conditions in participating patients.

## Conclusion

In summary, the rationale and design of a virtual care center that integrates first, second, and third line care into a virtual ward using remote monitoring and video consultation is presented.

## Data Availability Statement

The original contributions presented in the study are included in the article/supplementary material, further inquiries can be directed to the corresponding author/s.

## Author Contributions

AL, MS, SB, and RT: conceived and designed the experiments and wrote the paper. All authors contributed to the article and approved the submitted version.

## Conflict of Interest

RT reports receiving a speaker's fee from Boston Scientific, Pfizer, and Sanofi within the last 36 months. The remaining authors declare that the research was conducted in the absence of any commercial or financial relationships that could be construed as a potential conflict of interest.

## Publisher's Note

All claims expressed in this article are solely those of the authors and do not necessarily represent those of their affiliated organizations, or those of the publisher, the editors and the reviewers. Any product that may be evaluated in this article, or claim that may be made by its manufacturer, is not guaranteed or endorsed by the publisher.
